# Evaluation of Innate Immune Mediators Related to Respiratory Viruses in the Lung of Stable COPD Patients

**DOI:** 10.3390/jcm9061807

**Published:** 2020-06-10

**Authors:** Silvestro E. D’Anna, Mauro Maniscalco, Vitina Carriero, Isabella Gnemmi, Gaetano Caramori, Francesco Nucera, Luisella Righi, Paola Brun, Bruno Balbi, Ian M Adcock, Maria Grazia Stella, Fabio L.M. Ricciardolo, Antonino Di Stefano

**Affiliations:** 1 Istituti Clinici Scientifici Maugeri, IRCCS, Divisione di Pneumologia Telese, Via Bagni Vecchi 1, 82037 Benevento, Italy; silvestro.danna@icsmaugeri.it; 2 Dipartimento di Scienze Cliniche e Biologiche, AOU San Luigi Gonzaga, Orbassano (Torino), Università di Torino, Regione Gonzole 10, 10043 Torino, Italy; vitina.carriero@unito.it (V.C.); fabioluigimassimo.ricciardolo@unito.it (F.L.M.R.); 3 Divisione di Pneumologia e Laboratorio di Citoimmunopatologia dell’Apparato Cardio Respiratorio, Istituti Clinici Scientifici Maugeri SpA, Società Benefit, IRCCS, Veruno, Via Revislate 13, 28010 Novara, Italy; Isabella.gnemmi@icsmaugeri.it (I.G.); bruno.balbi@icsmaugeri.it (B.B.); antonino.distefano@icsmaugeri.it (A.D.S.); 4 Pneumologia, Dipartimento di Scienze Biomediche, Odontoiatriche e delle Immagini Morfologiche e Funzionali (BIOMORF), Università degli Studi di Messina, Piazza Pugliatti 1, 98122 Messina, Italy; gcaramori@unime.it (G.C.); dott.fnucera@gmail.com (F.N.); 5 Dipartimento di Oncologia, SCDU, Anatomia Patologica, AOU, San Luigi, Orbassano, Università di Torino, Regione Gonzole 10, 10043 Torino, Italy; luisella.righi@unito.it; 6 Dipartimento di Medicina Molecolare, Sezione di Istologia, Università di Padova, Via Ugo Bassi 58b, 35121 Padova, Italy; paola.brun@unipd.it; 7 Airways Disease Section, National Heart and Lung Institute, Imperial College London, Dovehouse St, London SW3 6LY, UK; ian.adcock@imperial.ac.uk; 8 Unità Operativa di Medicina, Ospedale G. Giglio Cefalù, Contrada Pietrapollastra, Via Pisciotto, 90015 Palermo, Italy; mgstella2002@yahoo.it

**Keywords:** COPD pathology, COPD phenotypes, viral load, innate immune response, outcome, disability

## Abstract

**Background:** Little is known about the innate immune response to viral infections in stable Chronic Obstructive Pulmonary Disease (COPD). Objectives: To evaluate the innate immune mediators related to respiratory viruses in the bronchial biopsies and lung parenchyma of stable COPD patients. **Methods:** We evaluated the immunohistochemical (IHC) expression of Toll-like receptors 3-7-8-9 (TLR-3-7-8-9), TIR domain-containing adaptor inducing IFNβ (TRIF), Interferon regulatory factor 3 (IRF3), Phospho interferon regulatory factor 3 (pIRF3), Interferon regulatory factor 7 (IRF7), Phospho interferon regulatory factor 7 (pIRF7), retinoic acid-inducible gene I (RIG1), melanoma differentiation-associated protein 5 (MDA5), Probable ATP-dependent RNA helicase DHX58 (LGP2), Mitochondrial antiviral-signaling protein (MAVS), Stimulator of interferon genes (STING), DNA-dependent activator of IFN regulatory factors (DAI), forkhead box protein A3(FOXA3), Interferon alfa (IFNα), and Interferon beta (IFNβ) in the bronchial mucosa of patients with mild/moderate (*n* = 16), severe/very severe (*n* = 1618) stable COPD, control smokers (CS) (*n* = 1612), and control non-smokers (CNS) (*n* = 1612). We performed similar IHC analyses in peripheral lung from COPD (*n* = 1612) and CS (*n* = 1612). IFNα and IFNβ were assessed in bronchoalveolar lavage (BAL) supernatant from CNS (*n* = 168), CS (*n* = 169) and mild/moderate COPD (*n* = 1612). Viral load, including adenovirus-B, -C, Bocavirus, Respiratory syncytial Virus (RSV), Human Rhinovirus (HRV), Coronavirus, Influenza virus A (FLU-A), Influenza virus B (FLU-B), and Parainfluenzae-1 were measured in bronchial rings and lung parenchyma of COPD patients and the related control group (CS). **Results:** Among the viral-related innate immune mediators, RIG1, LGP2, MAVS, STING, and DAI resulted well expressed in the bronchial and lung tissues of COPD patients, although not in a significantly different mode from control groups. Compared to CS, COPD patients showed no significant differences of viral load in bronchial rings and lung parenchyma. **Conclusions:** Some virus-related molecules are well-expressed in the lung tissue and bronchi of stable COPD patients independently of the disease severity, suggesting a “primed” tissue environment capable of sensing the potential viral infections occurring in these patients.

## 1. Introduction

Inflammation plays a pivotal role in the pathogenesis of chronic obstructive pulmonary disease (COPD) [1,2,3]. A symbiotic relationship between the microbiota and the innate and adaptive immune host response has been hypothesized [4]. The microbiota diversity, balanced by immune host responses, might be involved in the protective responses developing in challenged patients [4,5]. The innate immune system recognizes the microbial pathogens through pattern-recognition receptors (PRRs), which detect the pathogen-associated molecular patterns (PAMPs) of bacterial, fungal, and viral origin [4,5]. This interaction induces a cascade of events generating inflammatory host responses and the activation of adaptive immune responses [4,5]. Toll-like receptors (TLRs) recognize various microbial components of bacteria, fungi, and viruses [6]. In particular, some TLRs such as TLR3, TLR7, TLR8, and TLR9 are specific for viral recognition. TLR3 recognizes double-stranded RNA (dsRNA) produced by most viruses inducing the synthesis of type I interferons (IFNα/β) [6]. TLR7 and TLR8 recognize guanosine or uridine-rich single-stranded RNA (ssRNA) from viruses [7,8,9], and TLR9 is involved in the viral A/D-and bacterial B/K-type CpG DNA recognition [10,11].

Viral infections or dsRNA activates interferon regulatory factor (IRF)3 via TIR domain-containing adaptor inducing IFNβ (TRIF) [12,13]. Viral-induced gene transcription of IFNα and IFNβ converge on the activation and phosphorylation of IRF3 and IRF7 [14,15,16,17,18] in most cell types, or on IRF7 for plasmacytoid dendritic cells [19]. Retinoic acid inducible gene (RIG)-I and melanoma differentiation-associated gene (MDA)5 have been identified as cytosolic receptors for viral RNAs and dsRNAs, inducing the activation of IRF3 and IRF7 followed by induction of type I IFN genes [20]. RIG-I and MDA5 can be negatively regulated by LGP2 (laboratory of genetics and physiology) (DHX58) competing with these molecules for engagement with viral RNAs [21], and LGP2 downregulates IFN-I production during infection by influenza A viruses in A549 and HeLa cells [22]. Alternatively, LGP2 potentiates IFN-I induction after viral dsRNA engagement, through cooperation with MDA5 [23]. LGP2 biological activity is hence the subject of debate. The mitochondrial antiviral signaling (MAVS) proteins, also known as IFNβ promoter stimulator-1 (IPS-1) and STING endoplasmic reticulum resident transmembrane protein [24], are considered adaptor molecules connecting the RIG-I or MDA5 viral-engaged molecules to downstream signaling and gene activation [25,26]. ZBP1 (DAI) is a cytosolic receptor binding DNA mediating an IRF3 dependent–TLR9 independent type I interferon response [27,28]. The transcription factor FOXA3 inhibits rhinovirus-induced IFN production, IRF3 phosphorylation and viral clearance in human bronchial epithelial cells [29].

Therefore, the expression of these molecules (TLR3, TLR7, TLR8, TLR9, TRIF, IRF3, pIRF3, IRF7, pIRF7, RIG1, MDA5, LGP2, MAVS, STING, DAI, FOXA3, IFNα, IFNβ) may reflect the anti-viral immune responses. Few studies to date have assessed the expression of these molecules in the lung of stable COPD patients.

The aim of this study was to investigate the expression of innate immune mediators and signaling pathways related to viral load in the bronchial mucosa, lung parenchyma, and bronchoalveolar lavage (BAL) of patients with stable COPD of differing severity and age-matched control subjects with normal lung function.

## 2. Methods

### 2.1. Subjects

We recruited 24 subjects with normal pulmonary function and 34 COPD patients (Table 1). All COPD patients and healthy controls who underwent bronchoscopy, bronchial biopsy, and BAL were recruited from the Respiratory Medicine Unit of the Istituti Clinici Scientifici Maugeri Institute of Veruno (Veruno, Italy) (archival material) and San Raffaele Institute of Cefalù (Palermo, Italy).

The definition of diagnosis and severity of COPD patients was according to COPD international guidelines [www.goldcopd.com] [30].

All COPD patients were stable without any exacerbation in the six months before bronchoscopy and none was under treatment with theophylline, antibiotics, antioxidants, mucolytics, and/or glucocorticoids in the month prior to the bronchial biopsy. Control subjects were volunteers or patients with normal lung function who underwent bronchoscopy for hemoptysis due to tongue base varix or for control after surgical treatment for traumatic trachea stenosis. The peripheral lung tissues were collected at the S. Luigi University Hospital of Orbassano (Torino) during lung resection for a solitary peripheral neoplasm and no patient was under regular treatment with glucocorticoids and/or bronchodilators.

Ethical committees of the Istituti Clinici Scientifici Maugeri Veruno (Novara) (CTS: p95), San Raffaele Institute, Cefalù (Palermo) (CE: up2017/3375E) and San Luigi Hospital, Orbassano (Torino) (CE: 151/int) approved the study. Written informed consent was obtained from each participant and lung specimens were obtained according to local ethics and technical committee guidelines.

### 2.2. Lung Function Tests and Volumes

Lung function tests and volumes were assessed by a spirometry (6200 Autobox Pulmonary Function Laboratory; Sensormedics Corp., Yorba Linda, CA, USA) as previously described according to guideline recommendations [2,30].

### 2.3. Fiberoptic Bronchoscopy, Collection, and Processing of Bronchial Biopsies

Bronchoscopy and bronchial biopsies were performed as previously described [2]. Briefly, four bronchial biopsy specimens were taken from segmental and subsegmental airways of the right lower and upper lobes using size 19 cupped forceps. At least two samples were embedded in Tissue Tek II OCT (Miles Scientific, Naperville, IL, USA), frozen within 15 min in isopentane pre-cooled in liquid nitrogen, and stored at −80 °C. The best frozen sample was then oriented and 6 μm thick cryostat sections were cut for immunohistochemical light microscopy analysis and processed as described below.

### 2.4. Collection and Processing of the Peripheral Lung Tissue

Twenty-four patients undergoing lung resection surgery for a solitary peripheral neoplasm were recruited. Twelve were smokers with normal lung function and twelve were smokers with COPD. None of the patients had undergone preoperative chemotherapy and/or radiotherapy and none had been treated with antibiotics or respiratory drugs in the month prior to surgery. Lung tissue processing was performed as previously described [30]. Briefly, two to four randomly selected tissue blocks were taken from the subpleural parenchyma of the lobe obtained at surgery, avoiding areas grossly invaded by tumor. Serial sections 4 µm thick were first cut and stained with hematoxylin-eosin (H&E) in order to visualize the morphology and exclude the presence of microscopically evident tumor infiltration. Samples were frozen in liquid nitrogen pre-cooled isopentane after embedding in OCT as for bronchial biopsy processing and used for cryostat sections preparations and immunostaining of some viral related antigens. Immunostainings of frozen sections were performed as reported for bronchial biopsies.

### 2.5. Immunohistochemistry Analysis on OCT-embedded Bronchial Biopsies and Peripheral Lung Tissue

Immune-histochemical methods with a panel of antibodies specific for inflammatory cells (CD4+, CD8+, CD68+, neutrophil elastase+) or molecules studied were applied to one cryostat section from each biopsy (Table S1) as previously described [30]. Antibody binding was demonstrated with secondary anti-mouse (Vector, BA 2000) or anti-rabbit (Vector, BA 1000) or anti-goat (Vector, BA 5000) antibodies followed by ABC kit AP AK5000, Vectastain and fast-red substrate (red color) or ABC kit HRP Elite, PK6100, Vectastain and diaminobenzidine (DAB) substrate (brown color).

In peripheral lung tissue, the immunostaining was performed as previously described [2]. In brief, the sections were stained with the primary antibodies as reported in Table S1. For the negative control slides, normal rabbit, goat or mouse non-specific immunoglobulins (Santa Cruz Biotechnology, Dallas, TX, USA) were used at the same protein concentration as the primary antibody. Control slides were included in each staining run using human tonsils or nasal polyps as positive control for all the immunostainings performed [30].

### 2.6. Scoring System and Quantification of Immunohistochemistry in the Bronchial Biopsies

Morphometric measurements were performed with a light microscope (Leitz Biomed, Leica Cambridge, UK). The immunostaining for all antigens studied was scored (from 0 = absence of immunostaining to 3 = extensive intense immunostaining) in the intact (columnar and basal epithelial cells) bronchial epithelium, as previously described [2]. The final result was expressed as the average of all scored fields performed in each biopsy. A mean ± SD volume of 0.700 ± 0.260 mm of epithelium was analyzed in COPD patients and control subjects. Immunostained cells in bronchial biopsies lamina propria were quantified 100 μm beneath the epithelial basement membrane in several non-overlapping high-power fields until the whole specimen was examined. The final result, expressed as the number of positive cells/mm^2^, was calculated as the average of all the cellular counts performed in each biopsy.

### 2.7. Scoring System for Immunohistochemistry in the Peripheral Lung Tissue

Staining analysis was performed as previously described [30]. Data were interpreted in blinded fashion with no prior knowledge of the clinic-pathologic parameters. A bronchiole was taken to be an airway with no cartilage and glands in its wall. To count the number of positive cells on the sections stained for RIG1, MDA5, LGP2, MAVS, STING, DAI, FOXA3, IFNα, IFNβ, the area of bronchiolar epithelium, bronchiolar lamina propria, alveolar septa, and alveolar macrophages to be studied was selected. Cells with nuclear immunostaining were counted in all consecutive, non-overlapping high-power fields, including all available bronchioles for each section stained. Results were expressed as scored (0–3) values for each immune-staining.

To quantify molecule expression in alveolar macrophages and alveolar septa, at least 20 high-power fields (hpf) of lung parenchyma were randomly selected for each section. At least 100 macrophages inside the alveoli were evaluated. Alveolar macrophages were defined as mononuclear cells with well-represented cytoplasm present in the alveolar spaces and not attached to the alveolar walls using a previously validated method [30]. Results were expressed as scored values (from 0 = absence of immune-staining to 3 = extensive intense immune-staining) for each immune-staining.

### 2.8. Collection and Processing of Broncho-Alveolar Lavage

BAL was performed from the right middle lobe using four successive aliquots of 50 mL of 0.9% NaCl. BAL cells were spun (500× *g*; 10 min) and washed twice with Hanks’ buffered salt solution (HBSS). Cytospins were prepared and stained with May-Grünwald stain for differential cell counts. Cell viability was assessed using the trypan blue exclusion method. BAL supernatants were aliquoted and stored at −80 °C before their use for the ELISA assays summarized in Table S2. These assays were performed according to the manufacturer’s instructions.

### 2.9. Extraction of RNA/DNA and qRT-PCR for Viral Load Quantification in Bronchial Rings and Lung Parenchyma

Genesig standard kits (genesig Standard kit handbook HB10.04.09, published date 13 November 2017), including positive and negative controls for each one of the viruses studied, were used for qRT-PCR analysis of Adenovirus-C, Adenovirus-B, Bocavirus, Respiratory Syncytial Virus (RSV), Rhinovirus (HRV), Coronavirus, Flu-A, Flu-B, Virus Parainfluenzae-1. Genesig’s manufacturer instructions were carefully followed for viral RT-PCR quantification. RT-PCR cycling conditions were: 95 °C for 5 min (PCR initial activation step); 40 amplification cycles of 95 °C for 5 s (denaturation) and 60 °C for 10 s (combined annealing/extension), followed by melting curve analysis to ensure the specificity of the PCR amplification. For each reaction, negative controls were run in triplicate, consisting of primers, PCR Mastermix, and sterile water instead of DNA template. Positive controls for each virus studied were provided by the Genesig standard kits used. Amplification, data acquisition, and cycle threshold (CT) values analysis were performed using the Rotor Gene Q software (Rotor-Gene Q Series Software 2.0.2). Data were expressed as copies/µg of bronchial rings or lung parenchyma from each patient studied. All the viral RT-PCR quantitations were performed at least twice in different experimental sets for each specimen used and the results were accepted only after confirmation by the second experimental set.

### 2.10. Statistical Analysis

The data are shown as mean ± standard deviation (SD) for functional data and median (range) or interquartile range (IQR) for morphologic data. Differences between groups were analyzed using analysis of variance (ANOVA) for functional data, followed by the unpaired t-test for comparison between groups. The Kruskal-Wallis test followed by the Mann-Whitney U test for comparison between groups was used to compare morphologic data. Spearman rank method was used to calculate correlation coefficients. A p value less than 0.05 was considered significant. Data analysis was performed using the Stat View SE Graphics program (Abacus Concepts, Inc., Berkeley, CA, USA).

## 3. Results

### 3.1. Clinical Characteristics of Subjects

Bronchial biopsies from 58 subjects were evaluated: 34 with stable COPD, 12 current or ex-smokers with normal lung function, and 12 non-smokers with normal lung function (Table 1). COPD patients were divided into two groups: mild/moderate (GOLD stage I–II, *n* = 16) and severe/very severe (GOLD stage III–IV, *n* = 18). No differences in smoking history were found among the four groups. Values of FEV_1_ (% predicted) and FEV_1_/FVC (%) differed significantly between total COPD patients and both control groups. Severe/very severe COPD patients also differed significantly from mild/moderate COPD patients (ANOVA: *p* < 0.0001 for FEV_1_% predicted and FEV_1_/FVC% values). Thirty-five percent (*n* = 12) of the total COPD patients and 25% (*n* = 3) of healthy smokers with normal lung function also had symptoms of chronic bronchitis. There was no significant difference in functional data comparing COPD patients with healthy smokers for the presence of chronic bronchitis.

In addition, we studied peripheral lung specimens from 12 stable COPD patients and 12 control smokers with normal lung function matched for age and smoking history (Table 2).

### 3.2. Measurement of Inflammatory Cells in the Bronchial Biopsies of COPD Patients

These data, obtained from stable COPD patients by immunohistochemistry, confirmed previously reported higher numbers of neutrophils and CD8+ cells in severe/very severe COPD (Table 3) [30]. COPD patients with chronic bronchitis had a similar number of neutrophils when compared with COPD patients without chronic bronchitis [30].

### 3.3. Immunohistochemistry of Innate Immune Mediators Related to Respiratory Viruses in Bronchial Biopsies

The relevant anti-viral (TLR3, TLR7, TLR8, TLR9, TRIF, IRF3, pIRF3, IRF7, pIRF7, RIG1, MDA5, LGP2, MAVS, STING, DAI, FOXA3, IFNα, IFNβ) immune responses were analyzed.

### 3.4. Immunohistochemistry in Bronchial Epithelium

We considered significant the molecular changes when multiple comparisons (Kruskal Wallis test (KW) followed by comparisons between groups (Mann-Whitney test (MW)) were both significant. Only slight variations were observed for the immune-expression of this group of molecules since the statistical analysis based on multiple comparisons (KW) was not significant for any of the molecules studied (Table 3). TLR3 was tendentially increased in severe/very severe COPD (KW: *p* = 0.063, MW: *p* = 0.019) compared to control smokers. IRF7 was tendentially decreased in mild/moderate COPD (KW: *p* = 0.077, MW: *p* = 0.011) and control smokers (MW: *p* = 0.044) compared to control non-smokers. PhosphoIRF7 (KW: *p* = 0.214, MW: *p* = 0.037) was tendentially increased in severe/very severe COPD compared to control non-smokers. MDA5 was tendentially decreased in mild/moderate COPD (KW: *p* = 0.064, MW: *p* = 0.017) compared to control non-smokers. IFNβ was also tendentially decreased in mild/moderate COPD (KW: *p* = 0.061, MW: *p* = 0.019) compared to control non-smokers (Table 3). No significant differences were observed for any of the other molecules studied (Table 3).

### 3.5. Immunohistochemistry in Bronchial Lamina Propria

Similar to our observations in bronchial epithelium, only slight variations were observed for immune-expression in this group of molecules since the statistical analysis based on multiple comparisons was not significant for any of the molecules studied in the lamina propria (Table 3). TLR3 (KW: *p* = 0.128, MW: *p* = 0.023) and TLR8 (KW: *p* = 0.108, MW: *p* = 0.033) were tendentially increased in severe/very severe COPD in comparison to control smokers. TLR9 was tendentially increased in mild/moderate COPD (KW: *p* = 0.211, MW: *p* = 0.032) compared to control smokers. MAVS was tendentially increased in mild/moderate COPD (KW: *p* = 0.136, MW: *p* = 0.027) and control smokers (MW: *p* = 0.036) compared to control non-smokers. No significant differences were observed for the other molecules studied (Table 3).

**Figure 1:**
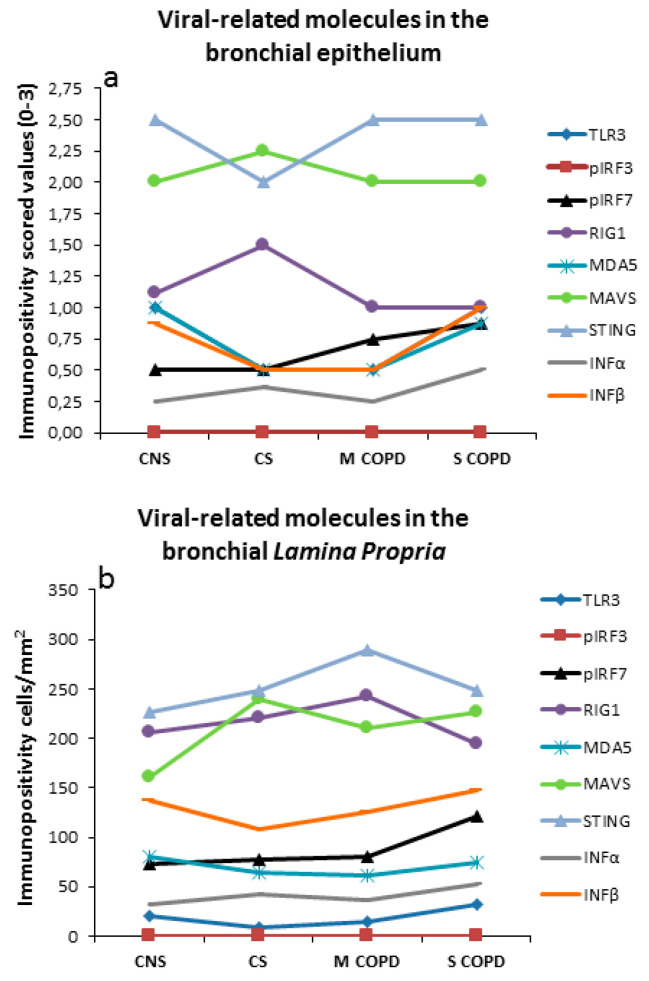
Schematic representation of the molecular variations related to anti-viral innate immunity response in the (**a**) bronchial epithelium and in the (**b**) bronchial lamina propria of control non-smokers, control smokers, mild/moderate stable COPD and severe/very severe stable COPD patients. Molecules involved in the anti-viral immune response are either poorly expressed (IFNα, IFNβ) or, when well expressed (RIG1, MDA5, MAVS, STING), values do not differ in the four groups of subjects studied. Range values for the molecules included in this graph are reported in Table 3. CNS = Control non-smokers; CS = Control smokers; MCOPD = Mild/moderate COPD; SCOPD = Severe/very severe COPD.

Figure 1 summarizes the principal molecules involved in the anti-viral immune response in the bronchial epithelium and in the bronchial lamina propria of COPD patients and control subjects.

### 3.6. Immunohistochemistry of Innate Immune Mediators Related to Respiratory Viruses in the Peripheral Lung Tissue

In the peripheral lung tissue of COPD patients (*n* = 12) and control smokers (*n* = 12) (Table 4), we measured semi-quantitatively the immune expression of RIG1, MDA5, LGP2, STING, DAI, FOXA3, IFNα, and IFNβ in bronchiolar epithelium, bronchiolar lamina propria, alveolar septa, and alveolar macrophages. No significant differences were observed between COPD patients and control smokers in all lung compartments considered for any of the molecules studied (Table 4).

### 3.7. ELISA Assays of Innate Immune Mediators Related to Respiratory Viruses in the BAL Supernatants

The clinical characteristics of the subjects for the BAL study are reported in Table 5. BAL levels of IFNα and IFNβ, the end-points of an immune anti-viral response, were measured. IFNα was under the detection limits of the method in almost all subjects studied. No significant difference was observed for IFNβ (median (range) (pg/mL): 0.44 (0–0.76); 0.46 (0–1.35) and 0.29 (0–1.38) (Kruskal Wallis, *p* = 0.767) respectively, for control non-smokers, control smokers, and mild/moderate COPD patients).

### 3.8. Correlations among Innate Immune Mediators Related to Respiratory Viruses, Clinical Parameters, and Inflammatory Cells in Bronchial Biopsies

No statistically significant correlations were found between viral-related innate immunity markers, inflammatory cells, or any other clinical parameters.

### 3.9. Viral Load in Bronchial Biopsies

Viral load in bronchial biopsies was not determined because of the lack of sufficient bronchial tissue for viral RNA/DNA extraction. Bronchial rings from surgical specimens of COPD patients (*n* = 12) and control smokers (*n* = 12) were used for viral quantitation in the large airways.

### 3.10. Viral Load in Bronchial Rings and Peripheral Lung Tissue

**Figure 2:**
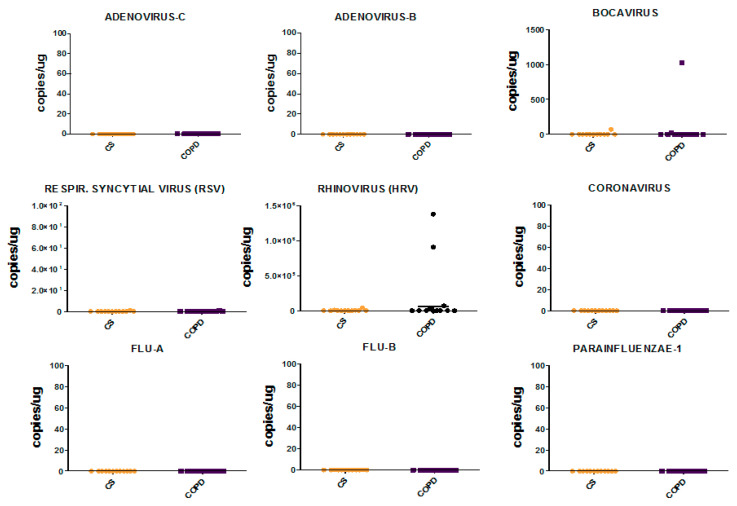
Viral load individual values in bronchial rings of control smokers (CS) and COPD patients quantified by RT-PCR. Horizontal bars are median values. Data are reported as copies/µg of bronchial ring tissue examined for Adenovirus-C, Adenovirus-B, Bocavirus, Respiratory Syncytial Virus (RSV), Rhinovirus (HRV), Coronavirus, Flu-A, Flu-B and Parainfluenzae-1. Results are representative of those from 12 subjects with stable COPD and 12 control smokers with normal lung function. The Mann-Whitney U test for statistical analysis was non-significant (*p* > 0.05) for all the viruses studied.

**Figure 3:**
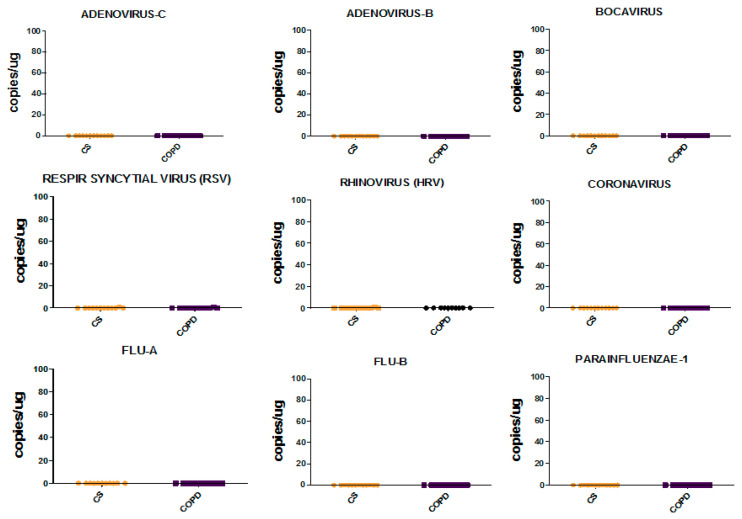
Individual values for viral load in lung parenchyma of control smokers (CS) and COPD patients quantified by RT-PCR. Horizontal bars are median values. Data are reported as copies/µg of lung parenchymal tissue examined for Adenovirus-C, Adenovirus-B, Bocavirus, Respiratory Syncytial Virus (RSV), Rhinovirus (HRV), Coronavirus, Flu-A, Flu-B and Parainfluenzae-1. Results are representative of those from 12 subjects with stable COPD and 12 control smokers with normal lung function. The Mann-Whitney U test for statistical analysis was non-significant (*p* > 0.05) for all the viruses studied.

Comparing COPD patients with control smokers (CS), we observed no significant differences for any of the viruses studied at both levels, bronchial rings and lung parenchyma (Figure 2 and Figure 3).

None of the viruses studied were found in the lung parenchyma of COPD and control smoking subjects. Rhinovirus was present at low levels in the bronchial rings of two COPD patients. Bocavirus was present at very low levels in the bronchial rings of one COPD patient and in one CS but it was not present in the peripheral lung. However, Bocavirus in the bronchial rings should be considered virtually absent, since 45 to 1000 copies/µg of tissue examined were quantified by RT-PCR analysis. Adenovirus-c and -b, RSV, Coronavirus, Flu-A, Flu-B and Parainfluenzae-1 viruses were not present neither in the bronchial rings nor in the lung parenchyma of COPD and CS subjects. None of the CS and COPD patients positive for Rhinovirus or Bocavirus presented symptoms related to this RT-PCR viral load quantitation in bronchial tissues.

## 4. Discussion

We have shown that some viral-related molecules, such as RIG1, MDA5, LGP2, STING, and DAI, are well expressed in the lung tissue and bronchi of both stable COPD at different stages of disease severity and control subjects, in the presence of low levels of respiratory viruses in both bronchial rings and lung parenchyma. On the other hand, the cytokines (IFNα, IFNβ) commonly involved in the anti-viral response are expressed at low levels in the bronchial mucosa, lung parenchyma, and in the BAL fluid of both stable COPD patients and control groups. We observed no significant differences for any of the viruses studied when comparing stable COPD patients and control smokers with normal lung function.

In contrast with the previously reported [31] anti-bacterial immune response, we observed only slight variations in the immune-expression of molecules involved in the anti-viral immune response, i.e., the statistical analysis based on multiple comparisons (Kruskal-Wallis test) was not significant for any of the anti-viral molecules studied in the bronchial epithelium or lamina propria of our COPD patients and control subjects. This was also true for the transcription factor FOXA3, an inhibitor of rhinovirus-induced IFN production [29], even though it was well expressed in the bronchial epithelium and lamina propria of all patients and control subjects studied. In addition, the viral-related molecules RIG1, MDA5, LGP2, STING, and DAI were well expressed in the bronchial and lung tissue of all patients and subjects studied, suggesting a “primed” state of the bronchial mucosa for viral clearance in these patients. Interestingly, the cytosolic receptor for viral RNAs MDA5 and the adaptor molecule STING mRNA levels measured in the bronchial biopsies were about 3-fold more abundant in severe/very severe COPD compared to control smokers, confirming a “primed” state of the bronchial mucosa for viral clearance in those patients. These intracellular viral sensors were then also analyzed in the peripheral lung specimens from mild/moderate COPD patients and control smokers, confirming that RIG1, MDA5, LGP2, MAVS, and STING, but not DAI viral cytoplasmic sensor, are well expressed in the different bronchiolar and lung compartments studied.

A progressive decrement of the total bacterial and viral load is reported from upper to lower airways and lung parenchyma [32,33,34,35] rendering more difficult the identification of differences between groups in the presence of very low levels of total bacterial or viral load, particularly in specimens from peripheral lung. This difference may also influence the related immune host response developing in the bronchi and lung tissue (different compartments) of COPD patients when compared to control subjects. From this point of view, bronchial biopsy analysis of microbiome and the related innate and adaptive immune host response may be more “sensitive” than peripheral lung tissue investigations. In line with this speculation, our observed RIG1, MDA5, LGP2, MAVS, and STING immune-expression in bronchial biopsies of COPD patients and CS was less evident in the peripheral airways. Furthermore, IRF7 in the bronchial epithelium was tendentially decreased in smokers with and without COPD compared to control non-smokers, probably exposing all smokers to the viral challenges. The difference in the immune-expression between bronchial biopsies and peripheral airways may also be influenced by: (a) the greater heterogeneity of the peripheral airways [32,33,34], (b) the more heterogeneous microbiome distribution in the lung parenchyma [32,34], and (c) anatomical differences in small vessels and capillary distribution [36,37].

In hospitalized patients’ multiplex PCR tests for respiratory viruses in nasopharyngeal swabs, bronchial aspirates and BAL fluid, 29.2% of samples tested positive and 60.2% of all viruses identified belonged to picornaviruses (rhinovirus or enterovirus) and influenza viruses [35]. The positivity rate reported for all respiratory viruses studied was higher in the upper than the lower respiratory tract [35].

Some authors have observed the presence of respiratory viruses in stable COPD patients with a wide range of prevalence ranging from 79.7% in Wilkinson et al. [38] to 16.2% in Seemungal et al. [39], 13.6% in Wilkinson et al. [40], 11.8% in McManus et al. [41], and 6.25% in Papi et al. [42]. This wide variability of viral presence could be due to the site of sampling: specimens from nasal aspirates in Seemungal et al. [39] vs. sputum samples in Wilkinson et al. [40], McManus et al. [41] and Papi et al. [42]. Other factors influencing the detection of the viruses could be the time of sampling from the last exacerbation or the use of antiviral drugs or corticosteroids (Table 6). Alternatively, we can hypothesize genetic factors or increased susceptibility to viral infections for these patients [43]. Interestingly, Utokapark et al. [44], studying specimens from lung resections of stage I COPD patients, showed the presence of influenza A and coronavirus 229E in 13 out of 20 COPD patients.

The difference between Utokapark et al.’s study and our data could partly be because the former study considered patients in stable condition at two weeks from the last exacerbation whereas our patients were at six months from last exacerbation. Differences in stable state of the disease and in the corticosteroid regimens used could in part explain the differences observed when comparing our present data with the cited literature. Another explanation could be due to a higher rate of vaccinations against influenza virus in our COPD > 65 years old population [45]. Other studies, analyzing sputum and nasal specimens, also showed a lack of viral load, in line with our findings [46,47], even though the selection criteria used differed, in part, from our study.

None of the individuals in the present study on viral load presented symptoms related to viral infections such as pneumonitis, exacerbation of chronic bronchitis, or gastroenteritis. Interestingly, in one study analyzing HRV by RT-PCR, including COPD patients with stable disease, 11/21 (35%) of the patients were asymptomatic [48], in line with our present data. Previous studies have shown that patients with asymptomatic infection to HRV have less intense inflammation together with lower peak viral titles [49,50]. We did not compare the degree of inflammatory markers in HRV positive vs. negative patients since only two of our COPD patients were positive for HRV in their bronchial rings and none were positive for HRV in the peripheral airways. In samples taken from outpatients, using multiplex PCR to test swab specimens for common respiratory viruses, 6.2–7% tested positive for at least one virus and over half of the infections in the adult population were classified as asymptomatic [51]. This raises the need for cutoff values for assays to determine when a respiratory virus is clinically significant in the different bronchial and lung samples studied (swab, sputum, BAL fluid, tissue) [51]. To our knowledge, at present, no current guidelines report cutoff values for respiratory viral quantitation in bronchial and lung tissue specimens of obstructive lung diseases. Our study may also contribute useful data for bronchial and peripheral lung tissue determinations in patients with stable COPD.

Our study has some limitations. Firstly, all our patients were in stable conditions and we do not know the effect of the viral-related innate immune response during a viral exacerbation. However, for ethical reasons and given the requirement of stable disease in patients selected for lung resection surgery, it is difficult to obtain bronchial biopsies or peripheral lung specimens in exacerbated COPD. Furthermore, we quantified the viral load in the lung tissue by RT-PCR analysis and viral-related molecules by a semi-quantitative approach in the peripheral lung. The use of different methods such as flow cytometry analysis may have provided additional information. Finally, different molecular patterns responsive to the viral challenges not included in the present study might also be involved.

In conclusion, some viral-related innate immune mediators are well-expressed in the bronchial and lung tissues of stable COPD patients, to a similar extent as in healthy controls, suggesting a “primed” tissue environment capable of sensing the potential viral infections occurring in these patients.

## Figures and Tables

**Table 1:** Clinical characteristics of COPD and control subjects who provided bronchial biopsies.

Groups	*n*	Age (years)	M/F	Pack Years	Ex/Current Smokers	FEV_1_ (% pred.) pre-β_2_	FEV_1_ (% pred.) post-β_2_	FEV_1_/FVC (%)
**Control non-smokers**	12	63 ± 13	10/2	0	0	117 ± 18	ND	86 ± 10
**Control smokers with normal lung function**	12	61 ± 7	9/3	43 ± 26	2/10	104 ± 13	ND	81 ± 6
**COPD stages I and II (mild/moderate)**	16	71 ± 8 ^§^	14/3	50 ± 28	6/11	63 ± 11 ^#^	67 ± 13	57 ± 9 ^#^
**COPD stages III and IV (severe/very severe)**	18	66 ± 9 ^§^	11/7	54 ± 36	13/5	35 ± 8 ^#^^,&^	38 ± 9	44 ± 10 ^#^^,&^

Patients were classified according to GOLD (http://www-goldcopd.com) levels of severity for COPD into: mild (stage I), moderate (stage II), severe (stage III), and very severe (stage IV). Data are mean ± SD. For COPD patients FEV_1_/FVC (%) are post-bronchodilator values. Abbreviations: M, male; F, female, FEV_1_: forced expiratory volume in one second; FVC, forced vital capacity; ND, not determined; COPD, chronic obstructive pulmonary disease. Statistics. (ANOVA)^#^, *p* < 0.0001, significantly different from control smokers with normal lung function and control never-smokers; ^&^, *p* < 0.0001, significantly different from mild/moderate COPD: (ANOVA)^§^, *p* < 0.05, significantly different from control smokers with normal lung function.

**Table 2:** Characteristics of COPD and control subjects who provided peripheral lung tissue for immune-histochemical study.

Groups	*n*	Age (years)	Sex M/F	Ex/Current Smokers	Pack-Years	Chronic Bronchitis (Yes/No)	FEV_1_ (% pred.)	FEV_1_/FVC%
**Control smokers**	12	70.1 ± 2	11/1	6/6	33.6 ± 9.7	no	101.8 ± 3.8	74.0 ± 2.0
**COPD patients**	12	68.1 ± 2	6/6	10/2	44.4 ± 24.4	no	86.9 ± 4.8 *	65.7 ± 2.6 ^&^

Abbreviations: COPD = chronic obstructive pulmonary disease; M: male; F: female; FEV_1_ = forced expiratory volume in one second; FVC = forced vital capacity. For COPD and control smoker subjects, FEV_1_% predicted and FEV_1_/FVC% are post-bronchodilator values. Data expressed as mean ± SEM; T-test: * *p* = 0.027; ^&^ *p* = 0.0035.

**Table 3:** Immunohistochemical quantification of inflammatory cells, innate immune molecules, and cytokines related to viral response in bronchial biopsies.

Epithelium (Score 0–3)	Control Non-Smokers	Control Smokers	Mild/Moderate COPD	Severe/Very Severe COPD	Kruskal Wallis *p* Value
TLR3	1.0 (0.12–1.5)	0.5 (0.12–1.5)	0.5 (0.12–1.25)	1.0 (0.12–2.5) ^&^	0.063
TLR7	0.75 (0.25–1)	0.75 (0.5–1.25)	0.5 (0.25–1)	0.75 (0.25–1.25)	0.373
TLR8	0.25 (0–0.5)	0.25 (0.12–0.5)	0.25 (0–0.75)	0.25 (0.12–1)	0.499
TLR9	0.5 (0.12–0.75)	0.37 (0.12–1)	0.5 (0–1)	0.5 (0.12–1.5)	0.851
TICAM (TRIF)	2 (1.5–2.5)	2.12 (1.5–2.75)	2 (0.75–2.5)	2 (0.75–2.75)	0.456
IRF3	0.25 (0–0.25)	0.25 (0–0.25)	0.25 (0–1)	0.25 (0–0.5)	0.406
Phospho-IRF3	0 (0–0)	0 (0–0)	0 (0–0)	0 (0–0)	n.d.
IRF7	1.5 (0.75–1.75)	0.75 (0.25–1.75) *	0.5 (0.25–1.5) *	0.75 (0.25–2.5)	0.077
Phospho-IRF7	0.5 (0.25–1)	0.5 (0–1.5)	0.75 (0.25–1.25)	0.87 (0.5–1) *	0.214
DDX58 (RIG1)	1.12 (0.5–2.5)	1.5 (0.5–2)	1 (0.5–2)	1 (0.5–2.5)	0.996
MDA5	1 (0.5–1.5)	0.5 (0.5–1.5)	0.5 (0.25–1.5) *	0.87 (0.25–1.5)	0.064
DHX58 (LGP2)	2 (1.5–2.5)	1.75 (0.75–2.5)	1.75 (1–2.5)	1.62 (1–2.5)	0.402
MAVS	2 (1.25–2.5)	2.25 (1.25–3)	2 (1–3)	2 (1–3)	0.472
STING (TMEM173)	2.5 (1.5–2.5)	2 (1.5–2.5)	2.5 (1.5–3)	2.5 (1.5–3)	0.208
DAI (ZBP1)	2 (1–2.5)	2.5 (1.5–3)	2 (1–2.5)	2.5 (1.5–3)	0.197
FOXA3	1.75 (0.5–2)	1.37 (0.5–1.5)	1.5 (0.75–2)	1.5 (0.5–3)	0.797
IFNα	0.25 (0–0.75)	0.37 (0–1)	0.25 (0.25–1)	0.5 (0.25–1.25)	0.169
IFNβ	0.87 (0.5–1.5)	0.5 (0.25–1.5)	0.5 (0.25–1) *	1 (0.5–1.5) ^§^	0.061
**Lamina Propria (cells/mm^2^)**					
TLR3	21 (4–64)	9 (0–48)	14 (4–85)	32 (0–263) ^&^	0.128
TLR7	99 (5–161)	95 (70–148)	120 (16–242)	123 (26–229)	0.557
TLR8	0 (0–43)	0 (0–5)	4 (0–41)	3 (0–21) ^&^	0.108
TLR9	4 (0–52)	5 (0–15)	13 (0–53) ^&^	4 (0–90)	0.211
TICAM (TRIF)	183 (117–348)	219 (145–312)	206 (71–425)	227 (129–387)	0.923
IRF3	17 (0–39)	13 (4–92)	24 (0–148)	13 (0–64)	0.541
Phospho-IRF3	0 (0–0)	0 (0–0)	0 (0–4)	0 (0–8)	0.594
IRF7	92 (45–193)	79 (28–210)	74 (24–206)	87 (32–348)	0.875
Phospho-IRF7	73 (40–204)	77 (0–203)	81 (32–272)	121 (40–366)	0.444
DDX58 (RIG1)	206 (118–274)	220 (77–272)	243 (168-403)	194 (114–281)	0.247
MDA5	81 (32–164)	65 (29–187)	62 (24–161)	75 (32–186)	0.908
DHX58 (LGP2)	303 (140–446)	335 (174–554)	343 (193–705)	330 (148–559)	0.897
MAVS	161 (51–226)	239 (55–426) *	210 (89–371) *	226 (60–521)	0.136
STING (TMEM173)	226 (86–400)	248 (51–460)	289 (97–435)	249 (161–511)	0.586
DAI (ZBP1)	202 (161–328)	254 (203–387)	290 (26–488)	267 (153–405)	0.237
FOXA3	188 (73–343)	173 (81–294)	135 (97–333)	175 (82–277)	0.650
IFNα	32 (9–118)	42 (6–87)	37 (8–312)	53 (11–161)	0.447
IFNβ	137 (74–258)	108 (43–276)	126 (52–290)	148 (78–287)	0.187
CD4	164 (101–212)	246 (37–500)	258 (107–731)	252 (66–470)	0.206
CD8	147 (76–301)	179 (86–657)	195 (86–523)	244 (111–355) *	0.365
CD68	284 (128–516)	275 (97–904)	367 (158–759)	340 (204–1054)	0.671
Neutrophil Elastase	93 (58–166)	97 (45–308)	94 (28–512)	151 (47–470) *^,&^	0.045

Abbreviations: COPD, chronic obstructive pulmonary disease. Data expressed as median (range); n.d. not determined. Statistics: The Kruskal-Wallis test was used for multiple comparisons followed by Mann-Whitney U test for comparison between groups: *, *p* < 0.05, significantly different from control non-smokers; ^&^ *p* < 0.05, significantly different from control smokers with normal lung function; ^§^ *p* < 0.05, significantly different from mild/moderate COPD. For the exact “*p*” values for comparison between groups, see Results section. We considered as “tendentially” significant the variations reported between groups (Mann-Whitney U test) since the multiple comparison (Kruskal-Wallis test) was not significant.

**Table 4:** Immunohistochemical quantification of selected viral-related innate immune molecules and cytokines in the peripheral lung.

Localization	Control Smokers with Normal Lung Function	COPD Patients	Mann-Witney U-Test *p* Value
**Bronchiolar epithelium (score 0–3)**			
**RIG1**	0.70 (0.78)	0.58 (0.36)	0.185
**MDA5**	0.96 (0.85)	0.97 (0.45)	0.689
**LGP2**	0.12 (0.12)	0.12 (0.05)	0.597
**MAVS**	1.4 (0.68)	0.75 (0.71)	0.224
**STING**	1.60 (0.50)	1.10 (0.77)	0.037
**DAI**	0.25 (0.05)	0.12 (0.02)	0.146
**FOXA3**	0.68 (0.81)	0.75 (0.46)	0.901
**IFNα**	0.30 (0.30)	0.42 (0.31)	0.435
**IFNβ**	0.25 (0.12)	0.33 (0.11)	0.123
**Bronchiolar submucosa (score 0–3)**			
**RIG1**	0.47 (0.44)	0.46 (0.25)	0.267
**MDA5**	0.50 (0.33)	0.50 (0.19)	0.666
**LGP2**	0.10 (0.10)	0.12 (0.00)	0.641
**MAVS**	0.5 (0.0)	0.25 (0.25)	0.068
**STING**	1.10 (0.25)	0.90 (0.50)	0.114
**DAI**	0.12 (0.05)	0.10 (0.32)	0.482
**FOXA3**	0.50 (0.25)	0.50 (0.12)	0.982
**IFNα**	0.38 (0.27)	0.43 (0.19)	0.544
**IFNβ**	0.11 (0.17)	0.17 (0.14)	0.538
**Alveolar septa** **(score 0–3)**			
**RIG1**	0.42 (0.23)	0.42 (0.24)	0.460
**MDA5**	0.50 (0.04)	0.50 (0.03)	0.758
**LGP2**	1.00 (0.50)	1.5 (0.75)	0.112
**MAVS**	0.5 (0.18)	0.62 (0.25)	0.324
**STING**	0.50 (0.25)	0.25 (0.00)	0.057
**DAI**	0.25 (0.25)	0.25 (0.05)	0.218
**FOXA3**	0.50 (0.05)	0.50 (0.02)	0.704
**IFNα**	0.40 (0.19)	0.43 (0.16)	0.885
**IFNβ**	0.25 (0.16)	0.25 (0.13)	0.644
**Alveolar macrophages (score 0–3)**			
**RIG1**	1.35 (1.12)	1.14 (0.42)	0.340
**MDA5**	1.14 (0.75)	1.46 (0.57)	0.460
**LGP2**	1.50 (0.25)	2.00 (0.75)	0.488
**MAVS**	0.5 (0.43)	0.62 (0.37)	0.853
**STING**	2.50 (0.50)	2.00 (1.00)	0.126
**DAI**	0.75 (0.93)	0.50 (0.50)	0.157
**FOXA3**	0.50 (0.31)	0.50 (0.43)	0.939
**IFNα**	1.40 (0.44)	1.33 (0.62)	0.839
**IFNβ**	0.62 (0.28)	0.80 (0.19)	0.084

Scored (0–3) data expressed as median and interquartile range (IQR)**.**

**Table 5:** Clinical characteristics of the subjects recruited for the Bronchoalveolar lavage (BAL) study.

	Control Non-Smokers	Control Smokers with Normal Lung Function	Mild to Moderate COPD
**Number**	8	9	12
**Age (years)**	66.9 ± 2.9	60.7 ± 3.3	71.7 ± 1.6 *
**Sex (M/F)**	1/7	9/0	9/3
**Ex/current smokers**	--	4/5	8/4
**Pack-years**	0	40.1 ± 6.6	60.6 ± 13.6
**Chronic bronchitis**	2	5	5
**FEV_1_% predicted**	106.5 ± 3.2	97.6 ± 2.8	59.9 ± 5.1 ^&^
**FEV_1_/FVC%**	81.9 ± 2.5	83.5 ± 2.8	54.8 ± 2.7 ^&^

Abbreviations: COPD = chronic obstructive pulmonary disease; M: male; F: female; FEV_1_ = forced expiratory volume in one second; FVC = forced vital capacity. FEV_1_% predicted and FEV_1_/FVC% are post-bronchodilator values. COPD patients were using short-acting inhaled β_2_-agonists (SABA) or short-acting inhaled antimuscarinics (SAMA) prn or regular long-acting inhaled β_2_-agonists (LABA) and/or regular inhaled anticholinergics, including SAMA or long-acting inhaled antimuscarinics (LAMA) at the dosage recommended in current COPD guidelines (www.goldcopd.org) at the time of their recruitment. Data expressed as means ± SEM; ANOVA test: * *p* < 0.01 compared to control smokers with normal lung function; ^&^ *p* < 0.0001 compared to control smokers with normal lung function and control non-smokers.

**Table 6:** Principal selection criteria and treatments used in some relevant studies.

Authors	Seemungal	Wilkinson 2006	Wilkinson 2017	Utokapark	Mc Manus 2007	Mc Manus 2008	Papi	Falsey
Weeks from last exacerbation	4	4	4	2	8	8	8–10	N.A
% patients treated with steroids	97 ICS	NA	NA	10 ICS	NA	100 ICS 9% Oral	97 ICS	67 ICS 20 Oral
Steroids/daily mean, Beclomethasone equivalent dosage	1120	1000	NA	NA	NA	948	980	NA
Oral steroids	No	No	No	NA	NA	Yes (9%)	No	NA

Legend: NA = Not Available; ICS = Inhaled corticosteroids.

## Supplementary Materials

The following are available online at https://www.mdpi.com/2077-0383/9/6/1807/s1, Table S1: Primary antibodies and immunohistochemical conditions used for identification of innate immune proteins, cytokines, and inflammatory cells, Table S2: Summary of the ELISA assays performed on the bronchoalveolar lavage (BAL) supernatants.
