# Supplementary material for: Evaluation of Innate Immune Mediators Related to Respiratory Viruses in the Lung of Stable COPD Patients
Source: J Clin Med. 2020 Jun 10;9(6):1807. doi: 10.3390/jcm9061807 (PMC7356645; doi:10.3390/jcm9061807)
Supplement: Supplementary file 1 [file jcm-09-01807-s001.pdf]

Journal of Clinical Medicine

MDPI

Supplementary materials

Table S1. Primary antibodies and immunohistochemical conditions used for identification of innate immune proteins, cytokines and inflammatory cells.

Target	Supplier	Cat.#[a]	Source	Dilution	Positive control
TLR3	Abcam	Ab59918	rabbit	1:50	Nasal polyp, tonsil
TLR7	Abcam	Ab45371	rabbit	1:150	Nasal polyp, tonsil
TLR8	Abcam	Ab53630	goat	1:250	Nasal polyp, tonsil
TLR9	Serotec	AHP1823	goat	1:150	Nasal polyp, tonsil
TICAM(TRIF)	Novus Biol.	NBP177221	rabbit	1:1200	Nasal polyp, tonsil
IRF3	Santa Cruz	Sc-376455	mouse	1:40	Nasal polyp, tonsil
Phospho-IRF3	Novus Biol.	NBP140619	rabbit	1:250	Nasal polyp, tonsil
IRF7	Santa Cruz	Sc-74472	mouse	1:80	Nasal polyp, tonsil
Phospho-IRF7	Bioss	Bs3196R	rabbit	1:500	Nasal polyp, tonsil
DDX58(RIG1)	Serotec	AHP1776T	rabbit	1:400<br>1:400(p)	Nasal polyp, tonsil
MDA5	Serotec	AHP2065	goat	1:150<br>1:100(p)	Nasal polyp, tonsil
DHX58(LGP2)	Serotec	AHP1777T	rabbit	1:400	Nasal polyp, tonsil
MAVS	Serotec	AHP1702T	rabbit	1:200	Nasal polyp, tonsil
STING(TMEM173)	Novus Biol.	NBP177900	rabbit	1:300	Nasal polyp, tonsil
DAI(ZBP1)	Serotec	AHP1780T	rabbit	1:200	Nasal polyp, tonsil
FOXA3	LS Bio	LS-B947	rabbit	1:100	Nasal polyp, tonsil
IFNα	Santa Cruz	Sc-17645	goat	1:100; 1:100(p)	Nasal polyp, tonsil
IFNβ	Santa Cruz	Sc-20107	rabbit	1:600; 1:300(p)	Nasal polyp, tonsil
CD4	Dako	M716	Mouse	1:100	Human tonsil
CD8	Dako	M7103	Mouse	1:200	Human tonsil
CD68	Dako	M814	Mouse	1:200	Human tonsil
Neutrophil elastase	Dako	M752	Mouse	1:100	Nasal polyp

[a]Cat#, catalogue number; (p): paraffin embedded peripheral lung tissue pretreated with citrate buffer (pH 6) and microwave exposure. See methods section for details.

Table S2. Summary of the ELISA assays performed on the bronchoalveolar lavage (BAL) supernatants.

Specificity	Manufacturer website	Catalogue code	Standard curve sensitivity range	BAL supernatants
**IFNα**	www.pblassaysci.com	41105-1	0-500 pg/mL	Concentrated x20 with Vivaspin
**IFNβ**	www.pblassaysci.com	41410-1A	0-2000 pg/mL	Concentrated x20 with Vivaspin

References

1. 1. Di Stefano A, Caramori G, Barczyk A, et al. Innate immunity but not NLRP3 inflammasome activation correlates with severity of stable COPD. Thorax. 2014;69(6):516-524.
2. 2. Vallese D, Ricciardolo FL, Gnemmi I, et al. Phospho-p38 MAPK expression in COPD patients and asthmatics and in challenged bronchial epithelium. Respiration. 2015;89(4):329-342.
